# Improvement of gemcitabine sensitivity of *p53*-mutated pancreatic cancer MiaPaCa-2 cells by *RUNX2* depletion-mediated augmentation of TAp73-dependent cell death

**DOI:** 10.1038/oncsis.2016.40

**Published:** 2016-06-13

**Authors:** M Nakamura, H Sugimoto, T Ogata, K Hiraoka, H Yoda, M Sang, M Sang, Y Zhu, M Yu, O Shimozato, T Ozaki

**Affiliations:** 1 Laboratory of DNA Damage Signaling, Chiba Cancer Center Research Institute, Chiba, Japan; 2 Laboratory of Cancer Genetics, Chiba Cancer Center Research Institute, Chiba, Japan; 3 Department of Regenerative Medicine, Graduate School of Medicine, University of Toyama, Toyama, Japan; 4 Research Center, Fourth Hospital of Hebei Medical University, Shijiazhuang, Hebei province, P.R. China; 5 Department of Urology, First Hospital of China Medical University, Shenyang, Liaoning Sheng province, P.R. China; 6 Department of Laboratory Animal of China Medical University, Shenyang, Liaoning Sheng province, P.R. China

## Abstract

Pancreatic cancer exhibits the worst prognostic outcome among human cancers. Recently, we have described that depletion of *RUNX2* enhances gemcitabine (GEM) sensitivity of *p53*-deficient pancreatic cancer AsPC-1 cells through the activation of TAp63-mediated cell death pathway. These findings raised a question whether *RUNX2* silencing could also improve GEM efficacy on pancreatic cancer cells bearing *p53* mutation. In the present study, we have extended our study to *p53*-mutated pancreatic cancer MiaPaCa-2 cells. Based on our current results, MiaPaCa-2 cells were much more resistant to GEM as compared with *p53*-proficient pancreatic cancer SW1990 cells, and there existed a clear inverse relationship between the expression levels of TAp73 and RUNX2 in response to GEM. Forced expression of TAp73α in MiaPaCa-2 cells significantly promoted cell cycle arrest and/or cell death, indicating that a large amount of TAp73 might induce cell death even in the presence of mutant p53. Consistent with this notion, overexpression of TAp73α stimulated luciferase activity driven by p53/TAp73-target gene promoters in MiaPaCa-2 cells. Similar to AsPC-1 cells, small interfering RNA-mediated knockdown of *RUNX2* remarkably enhanced GEM sensitivity of MiPaCa-2 cells. Under our experimental conditions, TAp73 further accumulated in *RUNX2*-depleted MiaPaCa-2 cells exposed to GEM relative to GEM-treated non-silencing control cells. As expected, silencing of *p73* reduced GEM sensitivity of MiPaCa-2 cells. Moreover, GEM-mediated Tyr phosphorylation level of TAp73 was much more elevated in *RUNX2*-depleted MiaPaCa-2 cells. Collectively, our present findings strongly suggest that knockdown of *RUNX2* contributes to a prominent enhancement of GEM sensitivity of *p53*-mutated pancreatic cancer cells through the activation of TAp73-mediated cell death pathway, and also provides a promising strategy for the treatment of patients with pancreatic cancer bearing *p53* mutation.

## Introduction

Human pancreatic cancer is a serious disease with 5-year survival rate of <5% and its incidence is increasing annually.^1, 2^ In this connection, pancreatic cancer is expected to be the second leading cause of cancer-related death by 2030.^3^ Although surgical resection is the preferred treatment for pancreatic cancer patients and it has been significantly improved, most cases are found at a late advanced unresectable stage. Nucleoside analog termed gemcitabine (GEM) has been used as a first-line standard chemotherapy for pancreatic cancer patients, however its efficacy is extremely limited.^4, 5^ To date, no validated biomarker is available that can allow the prediction of the prognostic outcome of the patients and also the treatment efficacy in pancreatic cancer. Therefore, a new attractive molecular target(s) for the early detection and the treatment of pancreatic cancer patients should be urgently required.

It has been well-established that tumor suppresser p53 has a critical role in tumor prevention.^6, 7^ Accumulating evidence strongly indicates that p53 is a nuclear transcription factor and transactivates numerous its target genes implicated in the induction of cell cycle arrest, cellular senescence and/or cell death in response to the exogenous as well as the endogenous stresses such as DNA damage.^8, 9^ Upon DNA damage, p53 is induced to accumulate in cell nucleus through the sequential post-translational modifications such as phosphorylation as well as acetylation and exerts its pro-apoptotic function.^10^ The amount of p53 is largely regulated at protein level. Under the physiological condition, p53 is kept at extremely low level through the interaction with a p53-specific E3 protein ubiquitin ligase MDM2, which subsequently targets p53 for ubiquitin-dependent degradation via the proteasome.^11^ When p53/MDM2 interaction is disrupted, p53 is rapidly stabilized in response to DNA damage.^9^ Recently, the additional E3 ubiquitin protein ligases including Pirh2, Trim24, COP1 and CHIP, which participate in the degradation of p53, have been identified.^12, 13^

Meanwhile, the extensive mutation search demonstrated that *p53* is frequently mutated in a variety of human cancer tissues.^14^ Over 90% of mutations are localized within the genomic region encoding its core sequence-specific DNA-binding domain, suggesting that the majority of p53 mutants lack the sequence-specific transactivation ability and pro-apoptotic function.^15^ Of note, *p53* is found to be mutated or lost in ~75% of pancreatic cancer.^16^ In contrast to the short-lived wild-type p53, mutant p53 has a longer half-life.^17, 18^ An increased stability of mutant p53 might be due to the interaction of mutant p53 with molecular chaperone HSP90, which has been shown to prevent mutant p53 degradation and thereby promoting its accumulation.^19^ In addition, Zheng *et al.*^20^ found that MDM2 isoforms prohibit MDM2-mediated degradation of mutant p53 and prolong its half-life. As described, ^21^ mutant p53 acts as a dominant-negative inhibitor against wild-type p53 and acquires pro-oncogenic potential. Indeed, mutant p53 is implicated in metastasis, resistance to anti-cancer drug and genomic instability.^22^

A small p53 family is composed of p53, p73 and p63. In a sharp contrast to *p53*, *p73* and *p63* are rarely mutated in human cancers.^23^ *p73* and *p63* encode two major isoforms such as transcriptionally active TA isoforms (TAp73 and TAp63) and N-terminally truncated ΔN ones (ΔNp73 and ΔNp63).^24, 25^ TA and ΔN isoforms are produced by alternative splicing and alternative promoter usage, respectively. As expected from their structural similarity, TA isoforms have an ability to transactivate overlapping set of p53-target genes and a pro-apoptotic function. Like p53, TAp73 and TAp63 are induced in response to a certain DNA damage.^26, 27^ By contrast, ΔN isoforms lose *N*-terminal transactivation domain and thereby lacking sequence-specific transactivation ability as well as pro-apoptotic function. Intriguingly, ΔN isoforms and mutant p53 display a dominant-negative behavior toward TA isoforms, and then acquire pro-oncogenic potential.^25^ Previously, it has been shown that deregulated expression of ΔNp63 stimulates pro-oncogenic β-catenin signaling pathway.^28^ Recently, Dolloo *et al.*^29^ demonstrated that ΔNp73 is able to induce the expression of angiogenic *VEGF-A* under tumor-relevant hypoxic condition. These observations indicate that ΔN isoforms might have their own target genes involved in carcinogenesis.

RUNX family, which is composed of RUNX1, RUNX2 and RUNX3, is a sequence-specific transcription factor and each of these family members has a distinct biological function. For example, *RUNX1* has been originally identified as a part of the chromosome translocation in acute myeloid leukemia and is involved in the establishment of the hematopoietic stem cells.^30, 31, 32^ In a sharp contrast to RUNX1, RUNX2 is absolutely required for the osteoblast differentiation and bone formation. As described,^33, 34^ *RUNX2*-deficient mice failed to form mineralized bone. For RUNX3, a growing body of evidence implies that RUNX3 is tightly linked to gastoric development and also acts as a tumor suppressor toward gastoric cancer.^35, 36^

Recently, we have found that depletion of *RUNX2* in *p53*-proficient human osteosarcoma-derived U2OS cells enhances adriamycin sensitivity in a p53/TAp73-dependent manner.^37, 38^ In addition, we have also described that GEM sensitivity is significantly improved in *RUNX2*-depleted *p53*-deficient human pancreatic cancer AsPC-1 cells in a TAp63-dependent fashion.^39^ These findings are consistent with the previous and recent reports showing that the expression level of *RUNX2* in a variety of human cancer tissues including pancreatic cancer is higher than that of their corresponding normal ones, and RUNX2 transactivates various target genes implicated in carcinogenesis, indicating that, in addition to osteogenesis, RUNX2 has an pro-oncogenic potential.^40^

In the present study, we have examined whether silencing of *RUNX2* in *p53*-mutated pancreatic cancer MiaPaCa-2 cells could enhance their GEM sensitivity. As p53 mutant acts as a strong dominant-negative inhibitor against wild-type p53, TAp73 and TAp63, it is important to adequately address this issue for the improvement of GEM efficacy in the presence of mutant p53.

## Results

### *p53*-mutated human pancreatic cancer MiaPaCa-2 cells exhibit GEM-resistant phenotype as compared with *p53*-proficient human pancreatic cancer SW1990 cells

**Figure 1:**
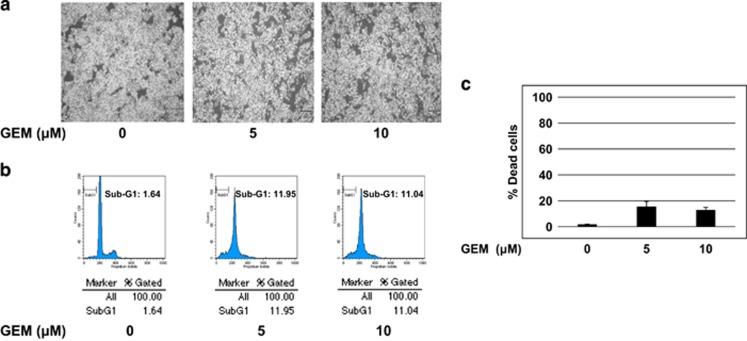
*p53*-mutated human pancreatic cancer MiaPaCa-2 cells are resistant to GEM. (**a**) Phase-contrast micrographs. MiaPaCa-2 cells were treated with the indicated concentrations of GEM. Forty-eight hours after treatment, representative pictures were taken. (**b, c**) MiaPaCa-2 cells undergo cell death following GEM exposure but to a lesser degree. MiaPaCa-2 cells were treated as in (**a**). Forty-eight hours after treatment, floating and attached cells were harvested and processed for FACS analysis (**b**) and trypan blue exclusion assay (**c**), respectively.

As mutant p53 acquires pro-oncogenic activity and also contributes at least in part to drug resistance of aggressive cancers, we have sought to examine GEM sensitivity of *p53*-mutated human pancreatic cancer MiaPaCa-2 cells and *p53*-proficient human pancreatic cancer SW1990 cells. For this purpose, both cells were treated with the indicated concentrations of GEM. Forty-eight hours after treatment, cells were observed under phase-contrast microscope, analyzed by flow cytometry and trypan blue exclusion assay. For SW1990 cells, number of the attached cells was remarkably decreased and cells clearly underwent cell death in response to GEM (Supplementary Figure S1). For MiaPaCa-2 cells, number of the adherent cells remained almost unchanged regardless of GEM exposure and GEM-mediated cell death took place but to a lesser degree (Figure 1). Therefore, these observations indicate that, like AsPC-1 cells,^39^ MiaPaCa-2 cells are much more resistant to GEM relative to SW1990 cells.

### Inverse relationship between the expression levels of TAp73 and RUNX2 in response to GEM

To understand the mechanistic basis of GEM-resistant phenotype of MiaPaCa-2 cells, we have checked the expression patterns of pro-apoptotic *p53* family members and their target gene products in response to GEM. In these experiments, the accumulation of γH2AX and the proteolytic cleavage of PARP following GEM exposure were examined by immunoblotting as a molecular marker for DNA damage and a mitochondrial dysfunction-mediated cell death, respectively.

**Figure 2:**
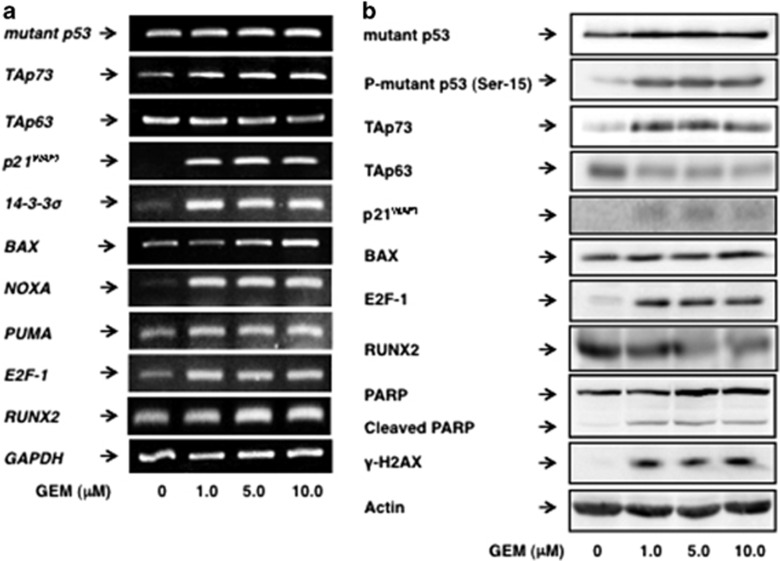
Inverse relationship between the expression levels of TAp73 and RUNX2 in response to GEM. MiaPaCa-2 cells were treated as in Figure 1a. Forty-eight hours after treatment, total RNA and cell lysates were prepared and analyzed by RT–PCR (**a**) and immunoblotting (**b**), respectively. *GAPDH* and actin were used as an internal control and a loading control, respectively.

As shown in Figure 2, GEM-mediated accumulation of γH2AX was clearly observed in MiaPaCa-2 cells, indicating that MiaPaCa-2 cells receive GEM-mediated DNA damage. However, GEM-induced decrease in the amount of the native PARP was barely detectable in MiaPaCa-2 cells. The expression level of transcriptionally active form of p73 termed TAp73 was increased in response to GEM (Supplementary Figure S2), which might be due to GEM-mediated up- and down-regulation of E2F-1 and RUNX2, respectively. According to the previous and recent observations,^38, 41, 42, 43^ E2F-1 and RUNX2 act as a transcriptional activator and a repressor of *TAp73*, respectively. Thus, there exists an inverse relationship between pro-apoptotic TAp73 and pro-oncogenic RUNX2 in MiaPaCa-2 cells following GEM exposure.

On the other hand, GEM treatment resulted in a massive reduction in another transcriptionally active p53 family member TAp63. At present, its functional significance is unclear. Consistent with the above-mentioned findings showing that TAp73 is induced to accumulate after GEM exposure, p53 family-target gene transcription such as *p21*^WAF1^, 14-3-3σ, *BAX*, *NOXA* and *PUMA*, was promoted in the presence of GEM. Considering that GEM displays a lower cytotoxic effect on MiaPaCa-2 cells, it is highly likely that mutant p53, which acts as a dominant-negative inhibitors against TAp73,^25^ weakens its pro-apoptotic ability. Intriguingly, forced expression of TAp73α in MiaPaCa-2 cells markedly promoted cell cycle arrest and/or cell death as examined by colony-formation assay (Supplementary Figure S3) and enhanced the luciferase activities driven by human *p21*^*WAF1*^ as well as *NOXA* promoter in a dose-dependent manner as examined by luciferase reporter analysis (Supplementary Figure S4). Thus, our observations strongly indicate that the intracellular balance between the amounts of mutant p53 and TAp73 has a vital role in the regulation of cell fate determination following GEM exposure.

For SW1990 cells, wild-type p53 was induced to accumulate and its phosphorylation level at Ser-15 was elevated after GEM exposure in association with the accumulation of γH2AX (Supplementary Figure S5). As expected, the amount of native PARP was significantly reduced following GEM treatment, implying that GEM-mediated induction of DNA damage triggers p53-dependent cell death in SW1990 cells. Under our experimental conditions, GEM treatment caused an increase in the transcription levels of *TAp73*, *TAp63*, *E2F-1* and *RUNX2*, whereas the amounts of their gene products markedly reduced following GEM exposure. At present, we do not know the molecular mechanisms behind this discrepancy.

### Knockdown of *RUNX2* enhances GEM sensitivity of MiaPaCa-2 cells

**Figure 3:**
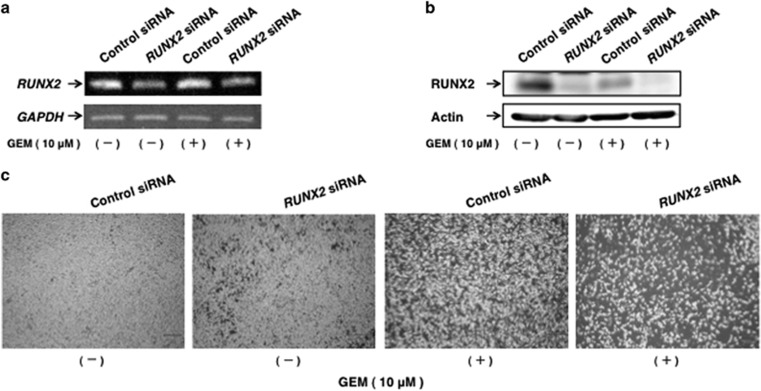
Depletion of *RUNX2* stimulates GEM-mediated reduction in number of viable cells. (**a, b**) siRNA-mediated silencing of *RUNX2*. MiaPaCa-2 cells were transfected with control siRNA or with siRNA targeting *RUNX2*. Twenty-four hours after transfection, cells were exposed to GEM (at a final concentration of 10 μm) or left untreated. Forty-eight hours after treatment, total RNA and cell lysates were extracted and subjected to RT–PCR (**a**) and immunoblotting (**b**), respectively. (**c**) Representative pictures.

Recently, we have found that depletion of *RUNX2* remarkably enhances the sensitivity to GEM of *p53*-deficient pancreatic cancer AsPC-1 cells.^39^ As shown above (Figure 2), the expression level of RUNX2 reduced in MiaPaCa-2 cells exposed to GEM. These findings prompted us to ask whether further down-regulation of *RUNX2* could improve the cytotoxic effect of GEM on MiaPaCa-2 cells. To this end, MiaPaCa-2 cells were transfected with control small interfering RNA (siRNA) or with siRNA against *RUNX2* (Smart pool siRNA mixture) followed by the incubation with or without GEM. Under our experimental conditions, siRNA-mediated knockdown of *RUNX2* was successful as examined by RT–PCR and immunoblotting (Figures 3a and b). Phase-contrast micrographs clearly showed that depletion of *RUNX2* results in a massive reduction in number of attached cells following GEM exposure relative to non-silencing control cells exposed to GEM (Figure 3c).

**Figure 4:**
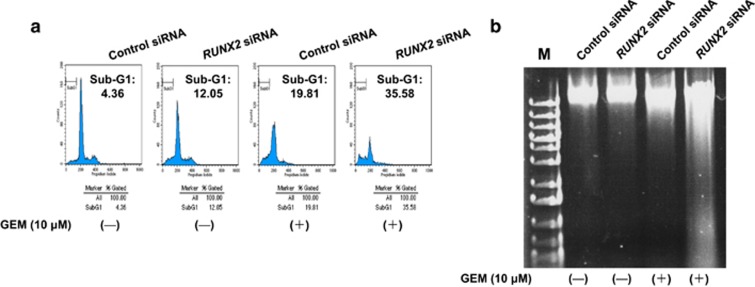
Knockdown of *RUNX2* enhances GEM sensitivity of MiaPaCa-2 cells. (**a**) FACS analysis. MiaPaCa-2 cells were treated as in Figure 3a. Forty-eight hours after treatment, floating and adherent cells were collected and analyzed by flow cytometry. (**b**) DNA fragmentation. MiaPaCa-2 cells were treated as in Figure 3a. Forty-eight hours after treatment, floating and adherent cells were collected and their genomic DNA was prepared according to the standard procedure. Genomic DNA was analyzed by 0.7% agarose gel electrophoresis and stained with ethidium bromide.

**Figure 5:**
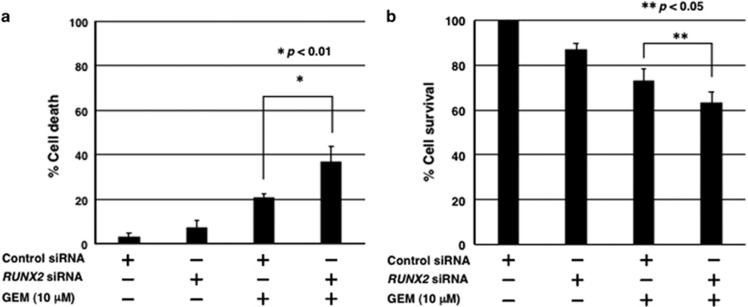
Depletion of *RUNX2* enhances the sensitivity to GEM of MiaPaCa-2 cells. (**a**, **b**) Effects of *RUNX2* knockdown on MiaPaCa-2 cells in the presence or absence of GEM. For trypan blue exclusion assay, MiaPaCa-2 cells were treated as in Figure 3a. Forty-eight hours after treatment, floating and attached cells were harvested and processed for trypan blue exclusion assay (**a**). For WST cell survival assay, MiaPaCa-2 cells were treated as in Figure 3a. Forty-eight hours after treatment, cells were subjected to WST cell survival assay (**b**). Results are presented as mean±s.d.

To further confirm the above-mentioned observations, non-depleted and *RUNX2*-depleted cells were treated with GEM or left untreated. Forty-eight hours after treatment, the attached and floating cells were collected and analyzed by flow cytometry. As shown in Figure 4a, around two-fold increase in number of cells with sub-G1 DNA content was detectable in *RUNX2*-depleted cells relative to non-depleted cells in the presence of GEM. Consistent with these observations, GEM-mediated DNA fragmentation was further stimulated in *RUNX2*-silencing cells as compared with that in non-silencing cells (Figure 4b). These results were also supported by the findings obtained from trypan blue exclusion and WST cell survival assays (Figures 5a and b). Similar results were also obtained in *RUNX2*-depleted MiaPaCa-2 cells mediated by two independent *RUNX2* siRNAs (Supplementary Figure S6). Together, our present results imply that silencing of *RUNX2* enhances GEM sensitivity of *p53*-mutated pancreatic cancer cells.

### Silencing of *RUNX2* augments TAp73-mediated cell death pathway

**Figure 6:**
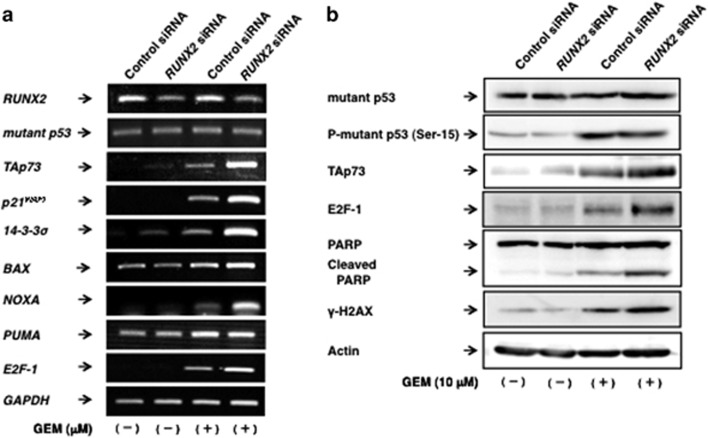
Silencing of *RUNX2* further stimulates GEM-mediated induction of TAp73 and cleavage of PARP. (**a, b**) Expression of TAp73 and its target genes in *RUNX2*-depleted MiaPaCa-2 cells in response to GEM. MiaPaCa-2 cells were treated as in Figure 3a. Forty-eight hours after treatment, total RNA and cell lysates were prepared and analyzed by RT–PCR (**a**) and immunoblotting (**b**), respectively.

To gain an insight into understanding the precise molecular mechanisms how knockdown of *RUNX2* could improve the efficacy of GEM on MiaPaCa-2 cells, non-silencing control cells and *RUNX2*-depleted cells were cultured in the presence or absence of GEM. Forty-eight hours after treatment, total RNA and cell lysates were prepared and analyzed by RT–PCR and immunoblotting, respectively. As expected, further stimulation of GEM-mediated *TAp73* transcriptional induction in *RUNX2*-depleted cells was observed as compared with non-depleted cells exposed to GEM (Figure 6a). Consistent with these observations, a massive induction of p53/TAp73-target genes such as *p21*^*WAF1*^, *14-3-3σ* and *NOXA* was detected in *RUNX2*-silencing cells in response to GEM. Similarly, immunoblotting experiments demonstrated that GEM-induced accumulation of TAp73 and cleaved PARP is obviously augmented by *RUNX2* knockdown (Figure 6b). As the further stimulation of GEM-mediated induction of E2F-1 was also detectable in *RUNX2*-silencing cells, it is likely that E2F-1 is tightly linked to the upregulation of TAp73 under our experimental conditions.

### Knockdown of *p73* reduces GEM sensitivity of MiaPaCa-2 cells

**Figure 7:**
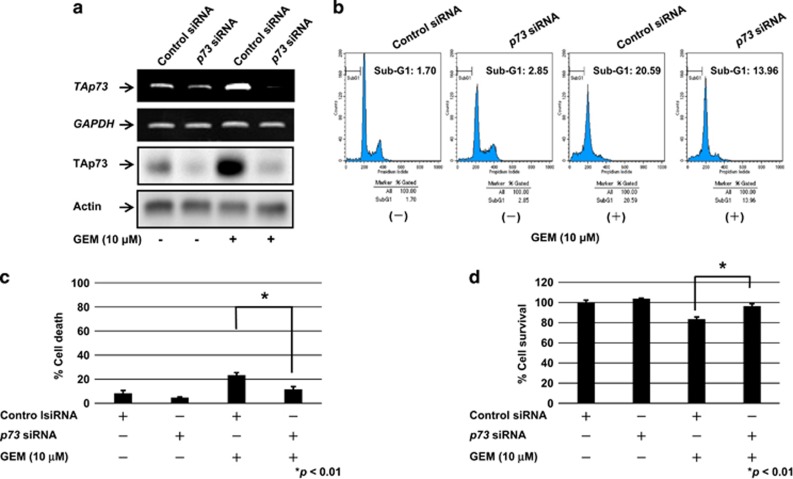
Depletion of *p73* reduces GEM sensitivity of MiaPaCa-2 cells. (**a**) siRNA-mediated knockdown of *p73*. MiaPaCa-2 cells were transfected with control siRNA or with siRNA against *p73*. Forty-eight hours after transfection, total RNA and cell lysates were prepared and analyzed by RT–PCR and immunoblotting, respectively. (**b**) FACS analysis. *p73*-depleted and non-depleted MiaPaCa-2 cells were exposed to GEM or left untreated. Forty-eight hours after treatment, floating and attached cells were harvested and subjected to flow cytometric analysis. (**c**, **d**) Trypan blue exclusion assay. MiaPaCa-2 cells were treated as in (**b**). Forty-eight hours after GEM exposure, floating and attached cells were collected and subjected to trypan blue exclusion assay.

To confirm our hypothesis that RUNX2/TAp73 regulatory axis could have a pivotal role in the modulation of GEM sensitivity of MiaPaCa-2 cells, MiaPaCa-2 cells were transfected with control siRNA or with siRNA targeting *p73*. As shown in Figure 7a, the endogenous TAp73 was successfully knocked down. Twenty-four hours after transfection, cells were treated with GEM or left untreated. Forty-eight hours after treatment, the attached and floating cells were collected and then subjected to the flow cytometric analysis. As seen in Figure 7b, GEM-mediated increase in number of cells with sub-G1 DNA content was attenuated by depletion of *p73*. In addition, trypan blue exclusion assay revealed that silencing of *p73* results in a significant decrease and an increase in number of dead and viable cells in response to GEM, respectively (Figures 7c and d). Thus, our observations indicate that TAp73 has a vital role in the regulation of GEM sensitivity of MiaPaCa-2 cells.

### GEM-mediated Tyr phosphorylation of TAp73 is augmented by *RUNX2* knockdown

**Figure 8:**
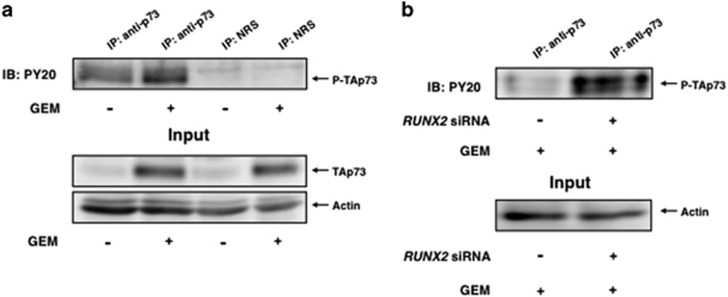
GEM-mediated Tyr phosphorylation of TAp73 is further stimulated in *RUNX2*-depleted MiaPaCa-2 cells. (**a**) GEM-mediated Tyr phosphorylation of TAp73. MiaPaCa-2 cells were treated with or without GEM. Forty-eight hours after treatment, cell lysates were prepared and immunoprecipitated with anti-p73 antibody or with normal rabbit serum (NRS). The immunoprecipitates were analyzed by immunoblotting with PY20. (**b**) GEM-mediated Tyr phosphorylation of TAp73 is augmented in *RUNX2*-depleted cells. MiaPaCa-2 cells were transfected with control siRNA or with siRNA against *RUNX2*. Twenty-four hours after transfection, cells were exposed to GEM for 48 h. After treatment, cell lysates were analyzed by co-immunoprecipitation experiments as in (**a**).

The next question to be solved is how *RUNX2* depletion could enhance the transcriptional and pro-apoptotic activities of TAp73 following GEM exposure. As described previously,^44, 45, 46^ TAp73 is stabilized and activated through c-Abl-mediated phosphorylation at Tyr-99 in response to DNA damage. These observations prompted us to examine whether GEM treatment could promote Tyr phosphorylation of TAp73 in MiaPaCa-2 cells. For this purpose, MiaPaCa-2 cells were treated with or without GEM for 48 h and then cell lysates were prepared. Cell lysates were then subjected to immunoprecipitation with anti-p73 antibody or with control IgG. The immunoprecipitates were finally analyzed by immunoblotting with PY20. As clearly shown in Figure 8a, TAp73 was induced to be phosphorylated at a certain Tyr residue(s) in response to GEM.

Subsequently, we sought to assess whether depletion of *RUNX2* could affect GEM-mediated Tyr phosphorylation of TAp73. Immunoprecipitation/immunoblotting experiments demonstrated that GEM-mediated Tyr phosphorylation level of TAp73 remarkably elevates in *RUNX2*-depleted MiaPaCa-2 cells relative to non-depleted MiaPaCa-2 cells (Figure 8b). Thus, these results suggest that RUNX2 attenuates GEM-dependent phosphorylation of TAp73 at Tyr residues, and thereby suppressing its pro-apoptotic activity.

## Discussion

Our recent findings strongly indicate that depletion of *RUNX2* improves the anti-cancer drug sensitivity of *p53*-proficient osteosarcoma U2OS cells as well as *p53*-deficient pancreatic cancer AsPC-1 cells in a p53 family-dependent manner.^37, 38, 39^ As described,^25^ mutant p53 exhibits a strong dominant-negative behavior against wild-type p53, TAp73 and TAp63, raising a question whether knockdown of *RUNX2* could also enhance the anti-cancer drug sensitivity of cancerous cells bearing *p53* mutation.

In the present study, we have found that silencing of *RUNX2* contributes to a significant enhancement of GEM sensitivity of *p53*-mutated pancreatic cancer MiaPaCa-2 cells through the augmentation of TAp73-mediated cell death pathway. As depletion of *RUNX2* improved adriamycin- and GEM sensitivity of U2OS cells and AsPC-1 cells, respectively,^37, 38, 39^ it is likely that knockdown of *RUNX2* has a vital role in the improvement of chemo-sensitivity of cancer cells regardless of their *p53* status.

Based on our current observations, MiaPaCa-2 cells were much more resistant to GEM as compared with *p53*-proficient SW1990 cells, which might be due to the presence of mutant p53. As described,^21, 24, 25^ mutant p53 displays a dominant-negative behavior against pro-apoptotic wild-type p53, TAp73 and TAp63. According to our results, the expression level of TAp73 was induced in MiaPaCa-2 cells exposed to GEM, however MiaPaCa-2 cells displayed a GEM-resistant phenotype. Therefore, it is possible that pro-apoptotic activity of TAp73 is weakened in the presence of a large amount of mutant p53 stably expressed in MiaPaCa-2 cells. For TAp63, its amount was markedly reduced at mRNA and protein level in GEM-exposed MiaPaCa-2 cells. At present, the precise molecular mechanisms behind GEM-mediated downregulation of TAp63 remain unknown. As MiaPaCa-2 cells underwent cell death in response to GEM but to a lesser degree, TAp63 might not contribute to GEM-mediated cell death observed in MiaPaCa-2 cells. Thus, it is suggestive that GEM sensitivity of MiaPaCa-2 cells might be determined at least in part by the intracellular balance between mutant p53 and TAp73 following GEM exposure.

As clearly shown in our colony-formation assay, forced expression of TAp73α significantly stimulated cell cycle arrest and/or cell death in MiaPaCa-2 cells. Consistent with these observations, overexpression of TAp73α in MiaPaCa-2 cells markedly enhanced the luciferase activities driven by human *p21*^*WAF1*^ and *NOXA* promoters in a dose-dependent manner. In support of our observations, it has been described that forced expression of TAp73 in *p53*-mutated cancerous cells results in a massive suppression of their proliferation rate.^47^ In addition, Muller *et al.*^48^ revealed that the intracellular balance between pro-apoptotic TAp73 and pro-oncogenic ΔNp73 is a critical determinant of chemo-sensitivity. Considering that mutant p53 acts as a dominant-negative inhibitor against TAp73,^25^ it is likely that, as mentioned above, a large amount of TAp73 might overcome the negative effect caused by mutant p53.

As shown in Figure 2, TAp73 was induced at mRNA and protein level following GEM exposure. Previously, it has been demonstrated that E2F-1 has as a transcriptional activator for *TAp73*, and E2F-1-mediated cell death is regulated at least in part in a TAp73-dependent manner.^41, 42, 43^ According to our present results (see Figure 6), knockdown of *RUNX2* in MiaPaCa-2 cells stimulated GEM-mediated increase in TAp73 in association with the further upregulation of E2F-1. As RUNX2 has an ability to suppress the expression of TAp73,^38^ RUNX2 might be also involved in the negative regulation of TAp73 as well as E2F-1 in response to GEM. Notably, Berman *et al.* found that loss of pRB, a strong inhibitor of E2F-1, promotes RUNX2 expression.^49^ Collectively, it is conceivable that there could exist a negative-feedback loop regulatory system where E2F-1 up-regulates its negative regulator RUNX2.

Unexpectedly, GEM treatment significantly reduced the expression level of RUNX2 protein, whereas its mRNA level was markedly increased in MiaPaCa-2 cells after GEM exposure. It has been shown that RUNX2 undergoes ubiquitin-mediated proteasomal degradation.^50^ Shen *et al.*^51^ revealed that E3 ubiquitin ligase Smurf1 (Smad ubiquitin regulatory factor 1) stimulates proteasome-mediated degradation of RUNX2, which is enhanced by Smad6. Distinct from Smurf1-mediated proteolytic degradation of RUNX2, it has been demonstrated that cyclin D1/Cdk4 complex phosphorylates RUNX2 and promotes its degradation in an ubiquitin/proteasome-dependent manner.^52^ In addition, several lines of evidence suggest that Smurf1 has a pro-oncogenic potential.^53, 54^ In a good agreement with this notion, Shain *et al.*^55^ described that *Smurf1* gene is amplified in certain subset of human pancreatic cancers and might contribute to their invasiveness. At present, it remains elusive whether Smurf1 could account for GEM-mediated degradation of RUNX2 in MiaPaCa-2 cells and contribute to the enhancement of their GEM sensitivity. Further studies should be required to adequately address this issue.

Another new finding of this study is that depletion of *RUNX2* in MiaPaCa-2 cells elevates GEM-mediated Tyr phosphorylation level of TAp73 and the accumulation of γH2AX (Supplementary Figure S7). As Tyr phosphorylation of TAp73 was detectable by immunoblotting with PY20 antibody, we did not know which Tyr residue(s) of TAp73 could be phosphorylated in response to GEM. According to the previous observations,^44, 45, 46^ TAp73 was phosphorylated at Tyr-99 by non-receptor type tyrosine kinase c-Abl following DNA damage, and the phosphorylated TAp73 became stabilized and activated. It has been well known that c-Abl is activated through ataxia telangiectasia mutated (ATM)-mediated phosphorylation after DNA damage.^56, 57^ Given that silencing of *RUNX2* further stimulates GEM-mediated accumulation of TAp73 as well as γH2AX, it is likely that TAp73 and/or RUNX2 participates in the regulation of DNA damage-dependent ATM phosphorylation. In accordance with this notion, we have recently found that TAp63 is tightly linked to GEM-mediated phosphorylation of ATM.^39^ Of note, Wang *et al.*^58^ indicating that c-Abl might act as an upstream regulator of ATM in response to DNA damage. Further studies should be required to clarify the functional interplay among p53 family, ATM/ATM Rad3-related protein, c-Abl and RUNX2 during DNA damage response. In addition, we have found putative Tyr phosphorylation sites other than Tyr-99 catalyzed by various protein tyrosine kinases including c-Abl and JAK within TAp73 amino-acid sequence (data not shown).

Taken together, our present results strongly suggest that depletion of *RUNX2* enhances GEM sensitivity of *p53*-mutated pancreatic cancer cells through the stimulation of TAp73-dependent cell death pathway, and thus RUNX2 might be an attractive molecular target for the treatment of the patients bearing pancreatic cancer regardless of *p53* status.

## Materials and methods

### Cell culture

The human pancreatic cancer SW1990 and MiaPaCa-2 cells were originated from the American Type Culture Collection (ATCC, Manassas, VA, USA). Cells were cultured in Dulbecco's Modified Eagle's medium supplemented with 10% (v/v) heat-inactivated fetal bovine serum (Life Technologies, Carlsbad, CA, USA) and penicillin–treptomycin in a 5% CO_2_ atmosphere at 37 °C.

### FACS analysis

For sub-G1 analysis, cells were exposed to GEM (Sigma–Aldrich, St Louis, MO, USA) at the indicated concentrations. Forty-eight hours after treatment, floating and adherent cells were collected, resuspended in phosphate-buffered saline (PBS) and fixed with ice-cold 70% ethanol for 15 min. After centrifugation, ethanol was removed and cells were rehydrated in PBS for 10 min. Cells were treated with RNAase A at a final concentration of 1 μg/ml and incubated for 30 min at 37 °C. Propidium iodide was subsequently added at a final concentration of 1 μg/ml and then analyzed by flow cytometry (FACS Calibur, BD Biosciences, Franklin Lakes, NJ, USA).

### WST cell survival assay

To examine cell viability, 1 × 10^3^ cells/well were seeded in a 96-well plate in 100 μl of culture medium. Twelve-hours after seeding, cells were treated with the indicated concentrations of GEM. Forty-eight hours after treatment, viable cells were counted using the Cell Counting Kit-8 (Dojindo, Kumamoto, Japan) following the manufacturer's instructions. Each experiment was carried out at least three times.

### Trypan blue exclusion assay

Cells were seeded at 2 × 10^5^ cells/six-well plate and allowed to attach overnight. Cells were then exposed to the indicated concentrations of GEM. Forty-eight hours after treatment, floating and adherent cells were collected and washed in PBS. After centrifugation, cells were resuspended in fresh medium, mixed with equal volume of 0.4% trypan blue solution and then analyzed by automatical cell counter (TC20, Bio-Rad, Hercules, CA, USA).

### Reverse transcription

Total RNA was isolated from the indicated cells using RNeasy Mini Kit (Qiagen, Valencia, CA, USA) according to the manufacturer's protocol. Reverse transcription was performed with 1 μg of total RNA using SuperScript II reverse transcriptase (Life Technologies) according to the manufacturer's instructions. The synthesized complementary DNA was amplified by PCR using specific primer sets. The PCR products were visualized by electrophoresis on agarose gels with ethidium bromide staining. *GAPDH* served as an internal control.

### Immunoblotting

Cells were washed twice in ice-cold PBS and lysed in lysis buffer containing 25 mM Tris-HCl, pH 7.5, 137 mM NaCl, 2.7 mM KCl, and 1% Triton X-100 and protease inhibitor cocktail (Roche Applied Sciences, Indianapolis, IN, USA). Protein concentration of cell lysates was determined by the Bradford assay (Bio-Rad). Equal amounts of cell lysates (50 μg of protein) were separated by 10% sodium dodecyl sulfate-polyacrylamide gel electrophoresis and transferred onto polyvinylidene difluoride membrane (Merck Millipore, Amsterdam, the Netherlands). The membrane was blocked with Tris-buffered saline containing 5% non-fat dry milk (Tris-buffered saline) and then incubated with anti-p53 (DO-1, Santa Cruz Biotechnology, Santa Cruz, CA, USA), anti-TAp73 (GeneTex, Irvine, CA, USA), anti-TAp63 (Cell Signaling Technology, Beverley, CA, USA), anti-p21^WAF1^ (H164, Santa Cruz Biotechnology), anti-BAX (Cell Signaling Technology), anti-E2F-1 (Cell Signaling Technology), anti-RUNX2 (Cell Signaling Technology), anti-PARP (Cell Signaling Technology), anti-γH2AX (2F3, BioLegend, San Diego, CA, USA) anti-ATM (5C2, Santa Cruz Biotechnology) or with anti-actin (20-33, Sigma–Aldrich) antibody. The membrane was washed in Tris-buffered saline-T and incubated with the appropriate horseradish peroxidase-conjugated secondary antibodies. After washing in Tris-buffered saline-T, immune-reactive signals were visualized with the enhanced chemiluminescence system (GE Healthcare Life Sciences, Piscataway, NJ, USA) according to the manufacturer's instructions.

### siRNA-mediated knockdown

MiaPaCa-2 cells were transfected with control siRNA or with a SMARTpool/ON-TARGETplus siRNA against *RUNX2* (Dharmacon, Lafayette, CO, USA) using Lipofectamine 2000 reagent (Life Technologies) following the manufacturer's protocols. Silencing of RUNX2 was evaluated by RT–PCR and immunoblotting.

### Immunoprecipitation assay

Cell lysates were prepared and incubated with mouse control IgG (Cell Signaling Technology) or with monoclonal anti-phospho-Tyr antibody (PY20, Abcam, Cambridge, MA, USA) overnight at 4 °C. Antibody-bound proteins were precipitated with protein G-Sepharose beads (GE Healthcare Life Sciences). The beads were washed three times in lysis buffer and then eluted in 2 × sodium dodecyl sulfate sample buffer. The eluted proteins were subjected to immunoblotting with anti-TAp73 antibody.

### Colony-formation assay

Cells were seeded at 2 × 10^5^ cells/six-well plate and allowed to attach overnight. Cells were transfected with the empty plasmid or with the expression plasmid for TAp73α. Forty-eight hours after transfection, cells were transferred to fresh medium containing 400 μg/ml of G418 (Sigma–Aldrich). Two weeks after the selection, G418-resistant colonies were fixed, stained with Giemsa's solution (Merck Millipore), air-dried and photographed.

### Luciferase reporter assay

Cells were seeded at 5 × 10^4^ cells/12-well plate and allowed to attach overnight. Cells were transfected with the constant amount of luciferase reporter plasmid bearing *p21*^*WAF1*^ or *NOXA* promoter and *Renilla* luciferase plasmid in the presence or absence of the increasing amounts of the expression plasmid for TAp73α. Total amount of plasmid DNA was kept constant by pcDNA3. Forty-eight hours after transfection, cell lysates were prepared and their luciferase activities were measured using Dual Luciferase Reporter System according to the manufacturer's instructions (Promega, Madison, WI, USA).

### Immunostaining

Cells were exposed to the indicated concentrations of GEM. Forty-eight hours after treatment, cells were fixed in 3.7% formaldehyde at room temperature for 30 min, permeabilized with 0.1% Triton X-100 at room temperature for 5 min and then blocked with 3% bovine serum albumin in PBS at room temperature for 1 h. After blocking, cells were washed in ice-cold PBS and incubated with mouse monoclonal anti-γH2AX antibody at room temperature for 1 h followed by the incubation with FITC-conjugated goat anti-mouse IgG (Life Technologies) at room temperature for 1 h. After the extensive washing, cell nuclei were stained with DAPI (Vector Laboratories, Peterborough, UK). Fluorescent images were captured using a confocal microscope.

### DNA fragmentation assay

*RUNX2*-depleted and non-depleted cells were exposed to GEM or left untreated. Forty-eight hours after treatment, floating and attached cells were harvested and their genomic DNA was extracted according to the standard procedure. One microgram of genomic DNA was analyzed by 0.7% agarose gel electrophoresis and visualized with ethidium bromide.

### Statistical analysis

All experiments were performed in triplicate. Data are presented as mean±s.d. Analysis utilized Student's *t*-tests and analysis of variance. Values of *P*<0.05 were considered significant.

## Abbreviations

ADR: adriamycin
ATM: ataxia telangiectasia mutated
ATR: ATM Rad3-related protein
DAPI: 4′,6-diamidino-2-phenylindole
DDR: DNA damage response
FACS: fluorescence-activated cell sorting
FITC: fluorescein isothiocyanate
GAPDH: glyceraldehyde 3-phosphate dehydrogenase
GEM: gemcitabine
PARP: poly ADP ribose polymerase
PI: propidium iodide
RUNX2: runt-related transcription factor 2
siRNA: small interfering RNA.
