# Supplementary figures and images for: Improvement of gemcitabine sensitivity of p53-mutated pancreatic cancer MiaPaCa-2 cells by RUNX2 depletion-mediated augmentation of TAp73-dependent cell death
Source: Oncogenesis. 2016 Jun 13;5(6):e233–. doi: 10.1038/oncsis.2016.40 (PMC4945741; doi:10.1038/oncsis.2016.40)

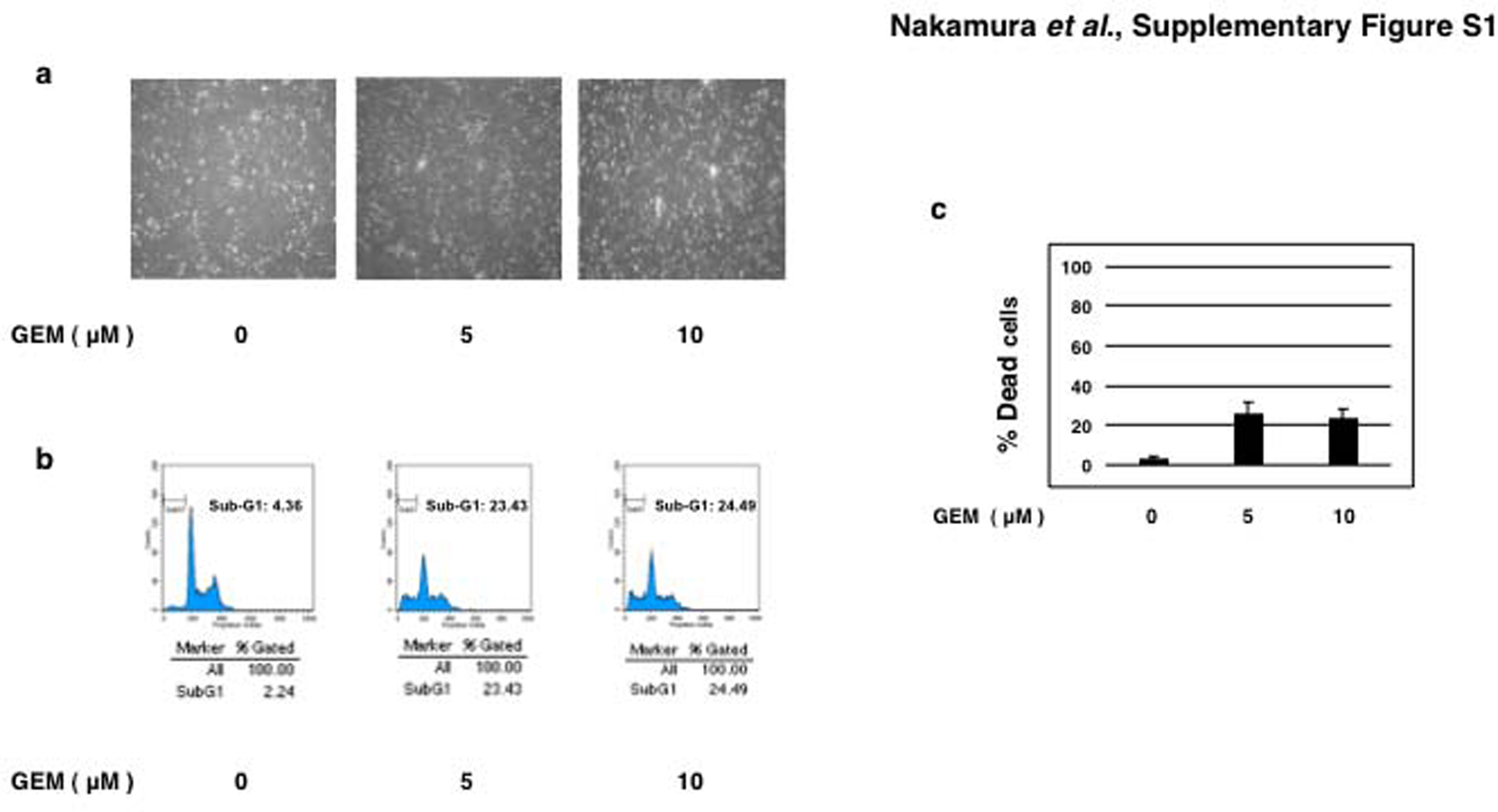

Supplement: Supplementary Figure 1 [file oncsis201640x1.tif]

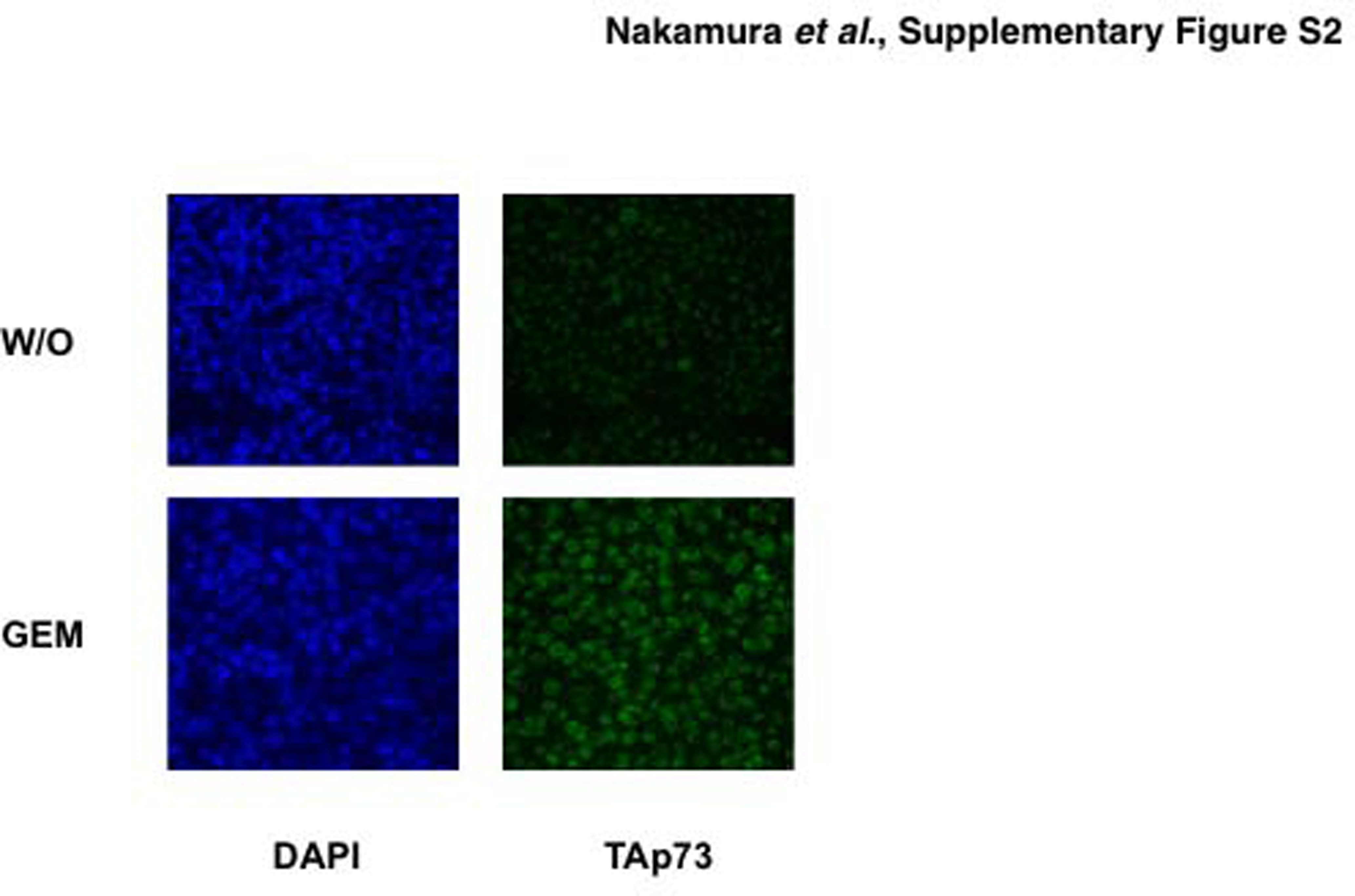

Supplement: Supplementary Figure 2 [file oncsis201640x2.tif]

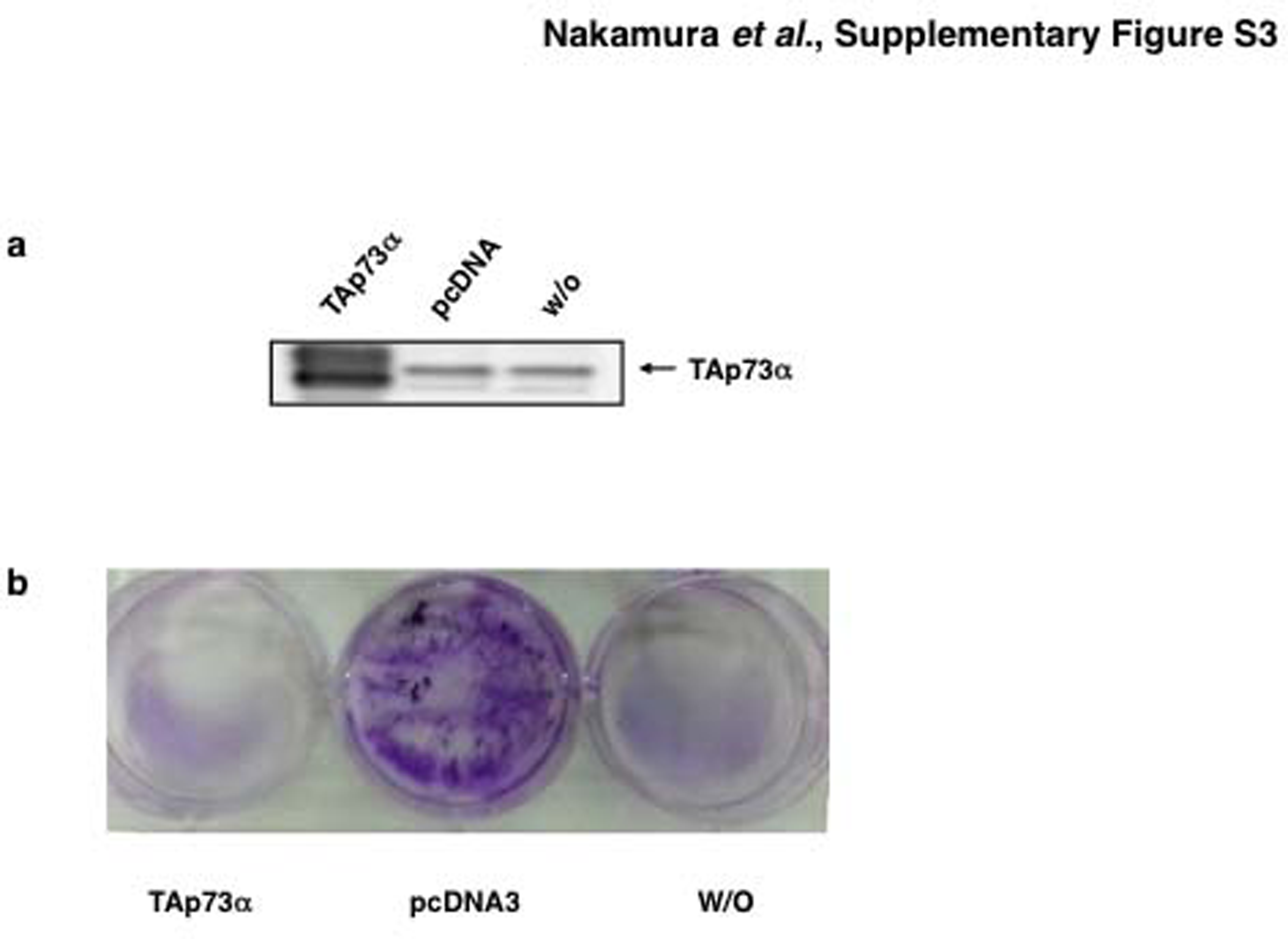

Supplement: Supplementary Figure 3 [file oncsis201640x3.tif]

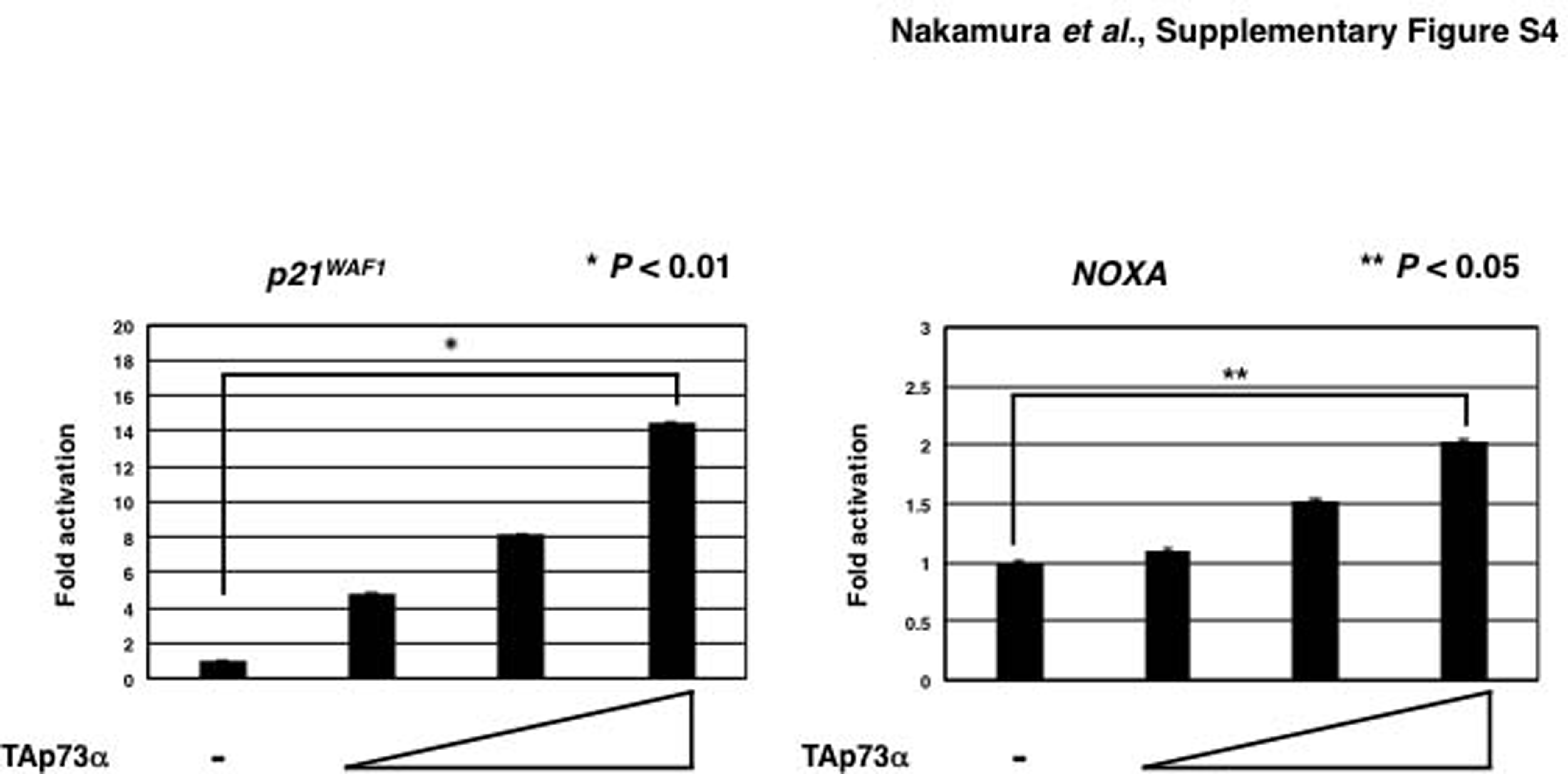

Supplement: Supplementary Figure 4 [file oncsis201640x4.tif]

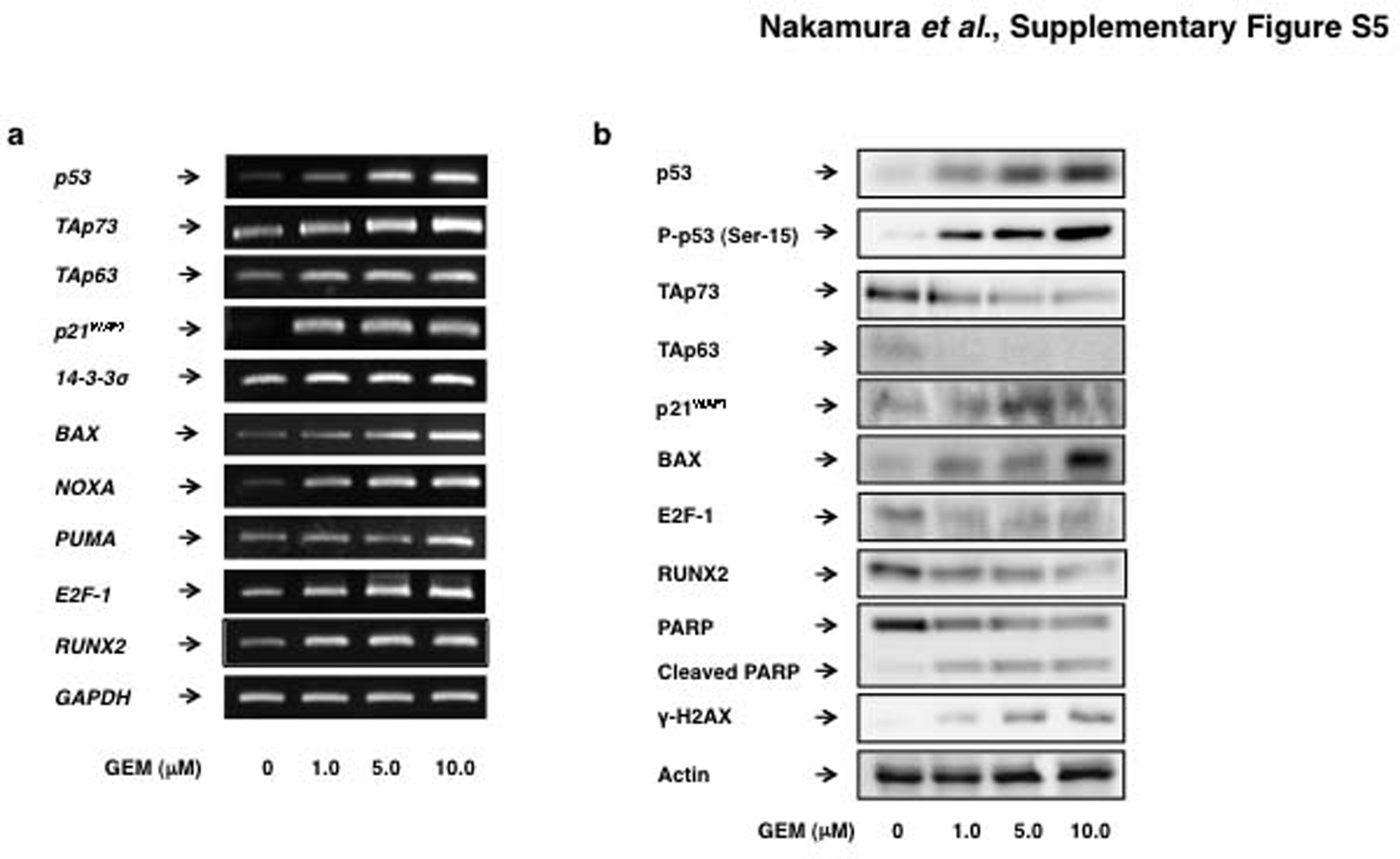

Supplement: Supplementary Figure 5 [file oncsis201640x5.tif]

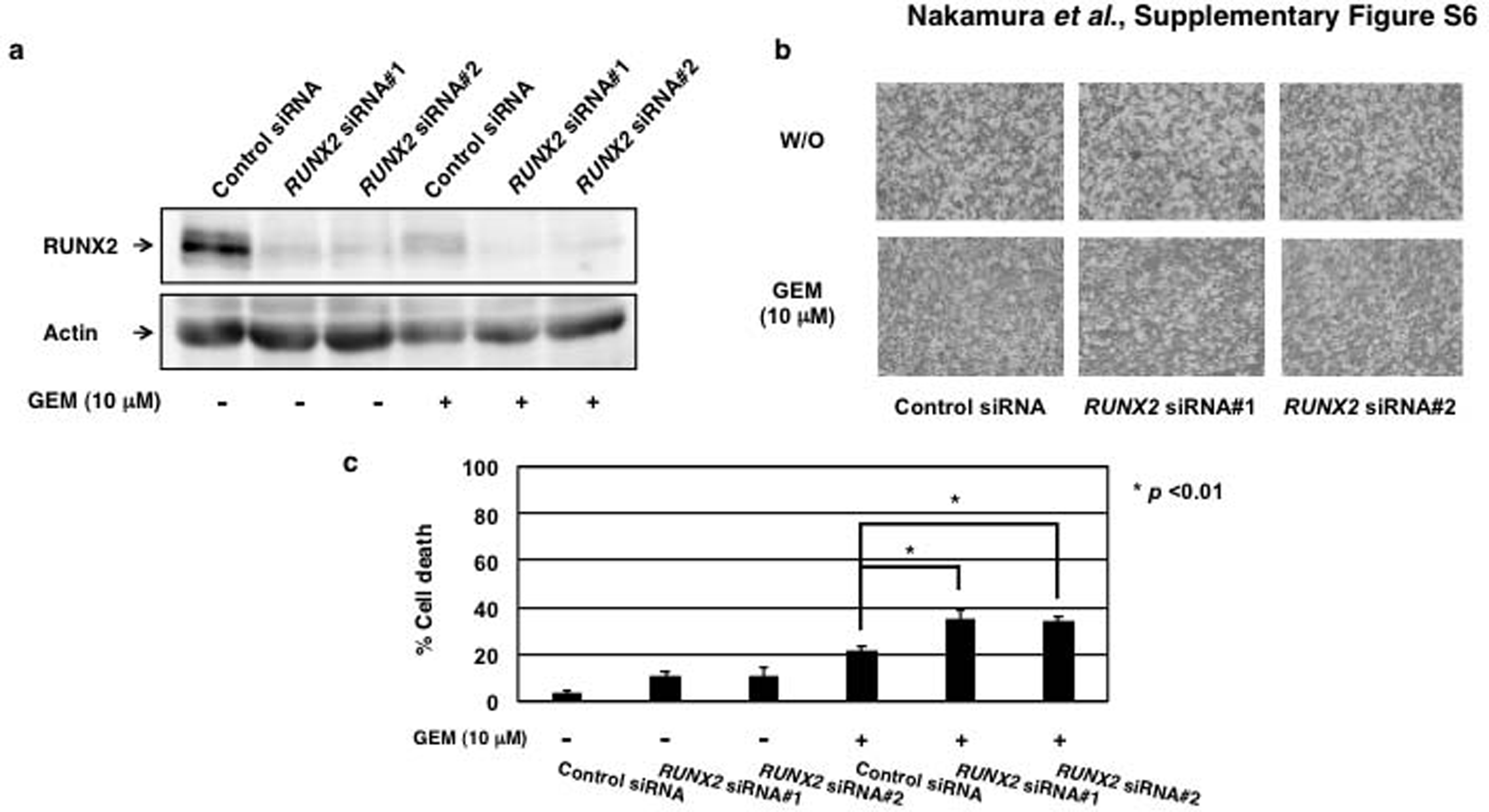

Supplement: Supplementary Figure 6 [file oncsis201640x6.tif]

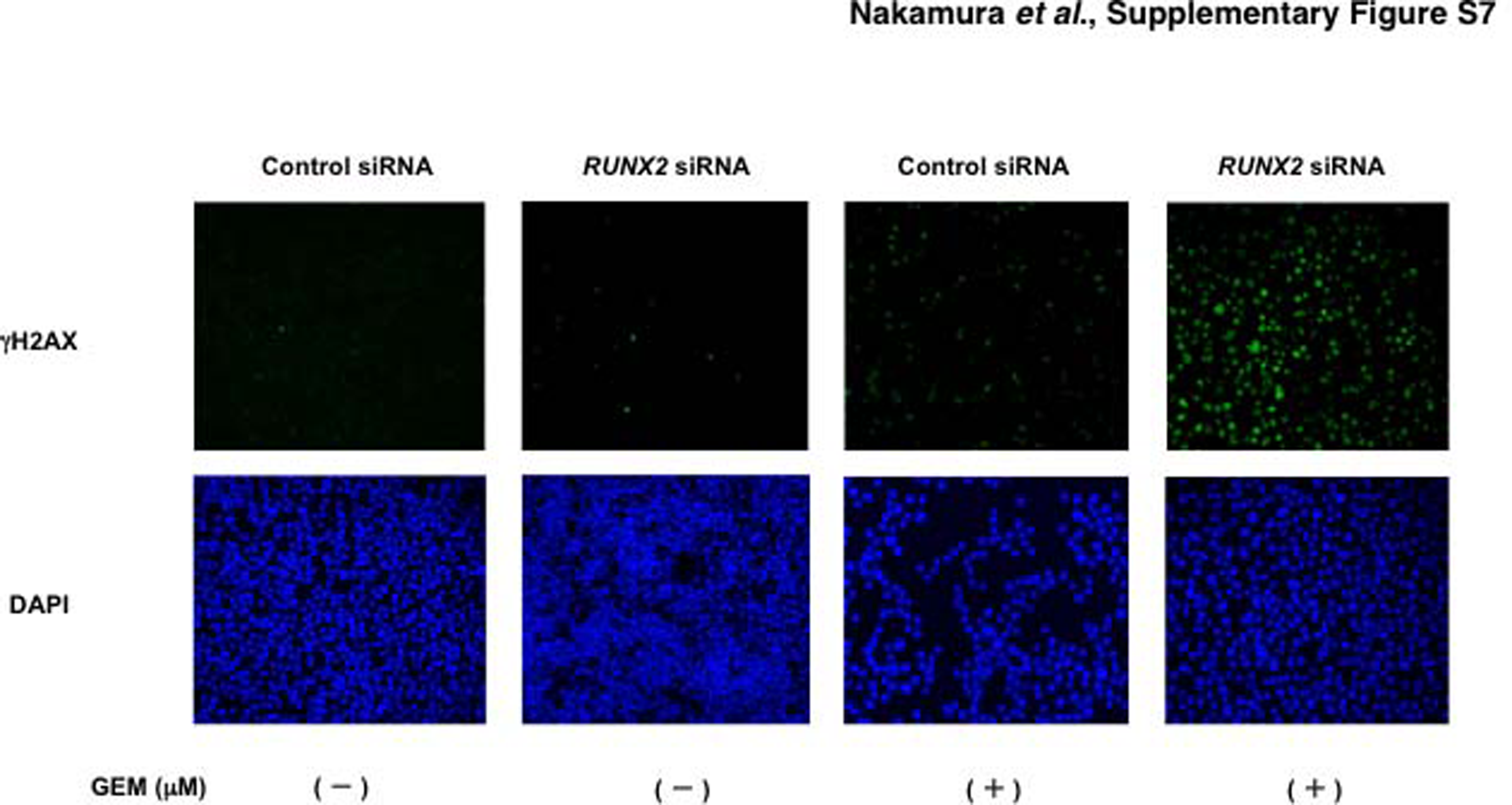

Supplement: Supplementary Figure 7 [file oncsis201640x7.tif]
