# Supplementary material for: Improvement of gemcitabine sensitivity of p53-mutated pancreatic cancer MiaPaCa-2 cells by RUNX2 depletion-mediated augmentation of TAp73-dependent cell death
Source: Oncogenesis. 2016 Jun 13;5(6):e233–. doi: 10.1038/oncsis.2016.40 (PMC4945741; doi:10.1038/oncsis.2016.40)
Supplement: Supplementary Information [file oncsis201640x8.docx]

**Supplementary Information**

**Figure Legends**

**Figure S1: *p53*-proficient human pancreatic cancer SW1990 cells are sensitive to gemcitabine (GEM).** (**a**) Phase-contrast micrographs. SW1990 cells were exposed to the indicated concentrations of GEM. Forty-eight hours after treatment, representative pictures were taken. (**b, c**) SW1990 cells undergo cell death in response to GEM. SW1990 cells were treated as in (**a**). Forty-eight hours after treatment, floating and attached cells were collected and subjected to flow cytometry (**b**) and trypan blue exclusion assay (**c**), respectively.

**Figure S2: Induction of TAp73 in response to GEM.** MiaPaCa-2 cells were exposed to 10 μM of GEM (lower panels) or left untreated (upper panels). Forty-eight hours after treatment, cells were fixed and incubated with anti-TAp73 antibody (green). Cell nuclei were stained with DAPI (blue).

**Figure S3: Forced expression of TAp73 promotes cell cycle arrest and/or cell death in MiaPaCa-2 cells.** MiaPaCa-2 cells were transfected with the expression plasmid for TAp73α or with the empty plasmid. Forty-eight hours after transfection, cell lysates were analyzed by immunoblotting (**a**). MiaPaCa-2 cells were transfected as in (**a**). Forty-eight hours after transfection, cells were transferred to the fresh medium containing G418 (at a final concentration of 400 μg/ml). Two weeks of selection, G418-resistant colonies were stained with Giemsa’s solution (**b**).

**Figure S4: Forced expression of TAp73 enhances luciferase activity driven by p53/TAp73-responsive promoters.** MiaPaCa-2 cells were transfected with the constant amount of the luciferase reporter plasmid bearing human *p21^WAF1^* or *NOXA* promoter and *Renilla* luciferase plasmid together with or without the increasing amounts of the expression plasmid for TAp73α. Total amount of plasmid DNA was kept constant with pcDNA3. Forty-eight hours after transfection, cell lysates were prepared and their luciferase activities were measured.

**Figure S5: SW1990 cells undergo cell death following GEM exposure in a p53-dependent manner.** SW1990 cells were treated as in Figure S1a. Forty-eight hours after GEM exposure, total RNA and cell lysates were prepared and analyzed by RT-PCR (**a**) and immunoblotting (**b**), respectively. For RT-PCR, *GAPDH* was used as an internal control. For immunoblotting, actin was used as a loading control.

**Figure S6: Silencing of *RUNX2* improves GEM sensitivity of MiaPaCa-2 cells.** MiaPaCa-2 cells were transfected with non-targeting control siRNA or with two independent siRNAs against *RUNX2* (siRNA#1 or siRNA#2), which are contained in Smart pool siRNA mixture (Dharmacon, Lafayette, CO, USA). Twenty-four hours after transfection, cells were treated with GEM (10 μM) or left untreated. Forty-eight hours after treatment, cell lysates were prepared and analyzed by immunoblotting (**a**). (**b**) Phase-contrast micrographs. MiaPaCa-2 cells were treated as in (**a**). Forty-eight hours after GEM exposure, representative pictures were taken. (**c**) MiaPaCa-2 cells were treated as in (**a**). Forty-eight hours after GEM treatment, floating and attached cells were harvested and analyzed by trypan blue exclusion assay.

**Figure S7: Depletion of *RUNX2* increases number of nuclear foci in response to GEM.** MiaPaCa-2 cells were transfected with control siRNA or with siRNA against *RUNX2*. Twenty-four hours after transfection, cells were treated with 10 μM of GEM or left untreated. Forty-eight hours after treatment, cells were fixed and probed with anti-γH2AX antibody (upper panels). Cell nuclei were stained with DAPI (lower panels).
